# Transitions to slow or fast diffusions provide a general property for in-phase or anti-phase polarity in a cell

**DOI:** 10.1007/s00285-020-01484-z

**Published:** 2020-03-20

**Authors:** S. Seirin-Lee, T. Sukekawa, T. Nakahara, H. Ishii, S.-I. Ei

**Affiliations:** 1 Department of Mathematics, School of Science, Hiroshima University, Higashi-hiroshima, 739-8530 Japan; 2 Department of Mathematics, Graduates School of Science, Hokkaido University, Sapporo, 060-0810 Japan; 3 Department of Mathematical and Life Sciences, Graduate School of Integrated Science for Life, Hiroshima University, Higashi-hiroshima, 739-8530 Japan; 4 JST PRESTO, 4-1-8 Honcho, Kawaguchi, Saitama 332-0012 Japan; 5 JST CREST, 4-1-8 Honcho, Kawaguchi, Saitama 332-0012 Japan

**Keywords:** 35Q92

## Abstract

Cell polarity is an important cellular process that cells use for various cellular functions such as asymmetric division, cell migration, and directionality determination. In asymmetric cell division, a mother cell creates multiple polarities of various proteins simultaneously within her membrane and cytosol to generate two different daughter cells. The formation of multiple polarities in asymmetric cell division has been found to be controlled via the regulatory system by upstream polarity of the membrane to downstream polarity of the cytosol, which is involved in not only polarity establishment but also polarity positioning. However, the mechanism for polarity positioning remains unclear. In this study, we found a general mechanism and mathematical structure for the multiple streams of polarities to determine their relative position via conceptional models based on the biological example of the asymmetric cell division process of *C. elegans* embryo. Using conceptional modeling and model reductions, we show that the positional relation of polarities is determined by a contrasting role of regulation by upstream polarity proteins on the transition process of diffusion dynamics of downstream proteins. We analytically prove that our findings hold under the general mathematical conditions, suggesting that the mechanism of relative position between upstream and downstream dynamics could be understood without depending on a specific type of bio-chemical reaction, and it could be the universal mechanism in multiple streams of polarity dynamics of the cell.

## Introduction

Cell polarity is an important cellular process that cells use for various cellular functions such as asymmetric division, cell migration, and directionality determination (Campanale et al. 2017). In particular, polarity formation in the asymmetric cell division process plays a core role in regulating the whole process of cell divisions occurred in early development of *C. elegans* embryo (Gönczy 2005). A mother cell divides its substrates asymmetrically into two daughter cells, which results in different gene expression between the daughter cells and leads to cells with different functions. To asymmetrically distribute the substrates, the mother cell creates a polarity pattern in the membrane that simultaneously induces polarities in the cytoplasmic proteins. After generating the polarities in both membrane and cytosol, a mother cell determines the cleavage plane via regulation by proteins forming polarities, which consequently determine the fate and size of the two daughter cells.

As a biological model system of asymmetric cell division, the polarity formation of *C. elegans* embryo has been well-studied. Before symmetry breaking, a group of transmembrane proteins, PAR-6, PAR-3, and PKC-3 [anterior PAR proteins (aPARs)], is homogeneously distributed in the membrane, and the other group of transmembrane proteins, PAR-2 and PAR-1 [posterior PAR proteins (pPARs)], is distributed homogeneously in the cytosol. Similarly, several cytoplasmic proteins homogeneously exist in the cytosol. MEX-5/6 and PIE-1 are the most well-studied cytoplasmic proteins that generate a polarity in the cytosol during asymmetric cell division. After the symmetry breaking induced by sperm entry as an external signal, aPARs and pPARs generate exclusive polarity domains of similar domain size in the membrane and determine the posterior and anterior axes in a single cell (Cuenca et al. 2002; Gönczy 2005). Interestingly, cytoplasmic proteins, MEX-5/6 and PIE-1, form polarity patterns in the cytosol simultaneously with PAR polarity formation in the membrane, but the location of polarity peaks are different between MEX-5/6 and PIE-1, where MEX-5/6 forms the polarity domain in the anterior end but PIE-1 forms a polarity domain in the posterior end (Daniels et al. 2010; Wu et al. 2015, 2018).

The exclusive domain formation of PAR proteins has been experimentally found to be underlined by the mutual inhibition interaction between aPARs and pPARs, in which the two protein groups transmit each other from the membrane to the cytosol via membrane binding/unbinding interaction (Hoege and Hyman 2013; Motegi and Seydoux 2013). In contrast, cytoplasmic polarities of MEX-5/6 and PIE-1 are based on the conversion of diffusion dynamics via a phosphorylation cycle (Daniels et al. 2010; Griffin et al. 2011; Tenlen et al. 2008; Wu et al. 2015, 2018). In particular, the phosphorylation cycles of MEX-5 are regulated by PAR proteins, and those of PIE-1 are regulated by MEX-5. Thus, PAR polarity is the upstream regulator of MEX-5/6 polarity, and polarity of MEX-5/6 is midstream regulator of PIE-1 polarity.

Theoretically, it has been well-studied that the underlying mechanism of PAR polarity formation is the bi-stability caused by the mutual inhibition effect of PARs in the membrane and the mass conservation property (Goehring et al. 2011; Seirin-Lee and Shibata 2015; Seirin-Lee 2016; Trong et al. 2014). A similar mathematical structure has been used to suggest most of the polarity formation mechanisms of the cell membrane (Jilkine and Edelstein-Keshet 2011; Mori et al. 2008; Otsuji et al. 2007). In contrast, cytoplasmic polarity has been poorly understood. In particular, the whole stream of polarity formation including both upstream of PAR polarity and midstream/downstream of cytoplasmic polarity has been very poorly understood by experiments, mathematical modeling, and analysis.

In this study, we focus on two questions. What is the essential mechanism or mathematical structure to generate the downstream polarity in a single cell with respect to the upstream polarity formation? Furthermore, what is the general mechanism by which the location of downstream polarity is determined with respect to the location of upstream polarity? As a solution, we constructed a conceptional mathematical model developed by the principal of capturing the essence of PAR, MEX-5/6, and PIE-1 dynamics. We then reduced the model more conceptually and introduce the two cytoplasmic polarity models. Using these models, we found that the positional relation between upstream and downstream polarity is determined by contrasting biochemical regulation by upstream protein on the conversion of diffusion dynamics of downstream proteins. We also found that the essential mechanism to create a cytosol polarity is independent of the mechanism to determine polarity position. We confirmed that the conceptional model is enough to understand the general mechanism of up–mid–down streams of polarity formation of PARs, MEX5/6, and PIE-1 in the asymmetric cell division process. Finally, we analytically proved that our findings hold under more general mathematical conditions. The results suggest that the mechanism of relative position between upstream and downstream dynamics could be understood without depending on specific bio-chemical reactions or phenomena, and it may be the universal mechanism for polarity positioning in multiple streams of polarity dynamics of a cell.

## Model formulation

### Conceptional model for upstream and downstream polarities in a cell

**Fig. 1:**
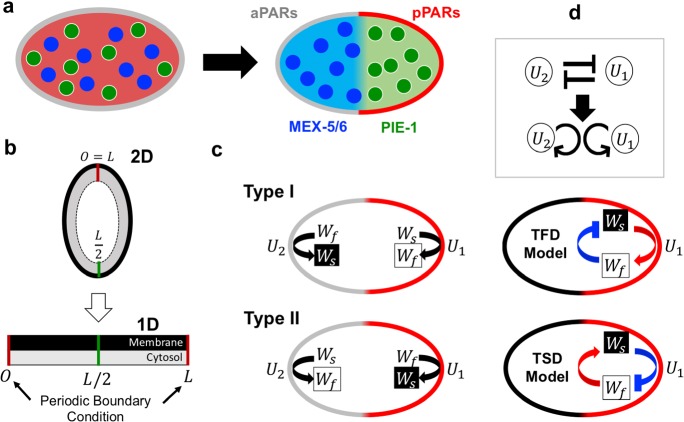
Schematic diagram of the model. **a** Polarity formation in *C. elegans* embryo. **b** Model simplification from two dimensional space to one dimensional space [0,  *L*]. **c**$U_1$ (red) and $U_2$ (gray) denote membrane proteins that generate each polarity exclusively. The thick arrows in the cell represent $W_s+U_{i} \xrightarrow {~\mu _1~} W_f+U_{i}$ or $W_f+U_{i}\xrightarrow {~\mu _2~} W_s+U_{i}$ ($i=1, 2$). **d** Transit to fast diffusion (TFD) and transit to slow diffusion (TSD) models with self-recruitment model reduction. The red arrows imply the activation effect, and the blue arrows denote the inhibition effect on the transition from slow/fast to fast/slow (color figure online)

In the *C. elegans* embryo, aPARs and pPARs in the membrane create mutually exclusive polarity. At the same time, a cytoplasmic protein MEX-5/6 biases toward the side where aPAPs generate a polarity (Fig. 1a). The upstream polarity of PARs can be generated spontaneously without the downstream polarity of MEX-5/6, but MEX-5/6 requires the existence of polarity of PARs (Cuenca et al. 2002). Based on the biological evidences for MEX-5/6 and PAR polarity dynamics, we consider a general type of upstream and downstream polarity model here. We formulate a top-to-down model where upstream polarity is not affected by downstream polarity to focus on the essential mechanism of downstream polarity formation and its polarity location in terms of upstream polarity.

Now, we assume two proteins that generate mutually exclusive polarity domains in the membrane by $U_1$ and $U_2$, respectively. Because we know that a reaction–diffusion model satisfying bi-stability and mass conservation can generate a polarity (Mori et al. 2008; Otsuji et al. 2007; Seirin-Lee and Shibata 2015), we choose the PAR polarity model suggested by Seirin-Lee and Shibata (2015), where $U_1$ and $U_2$ are transmembrane proteins that are mutually inhibited by translocating each other from the membrane to the cytosol. We also assume that the concentrations of transmembrane proteins, $U_1$ and $U_2$, in the cytosol approach steady states quickly between the peripheral area of cell membrane and the center area of cytosol, namely, well-mixed states in the bulk space of cytosol because the diffusion coefficients in the cytosol are higher than that in the membrane (Kuhn et al. 2011; Goehring et al. 2011). In addition, the imaging data of cytoplasmic proteins, *e.g.* MEX-5 and PIE-1, shows that the interfaces of protein distributions are almost linear along to the vertical-axis as shown in schematic figure of Fig. 1a (Cuenca et al. 2002; Daniels et al. 2010), indicating that capturing the the patterning dynamics in the periphery of cell membrane would be enough to understand the essential dynamics of polarity, in particular, the positioning of polarity. Because the reaction dynamics in this study is also based on the membrane binding/unbinding kinetic, we consider the cell domain in a one dimensional space [0, *L*] with periodic boundary conditions (Fig. 1b).

In what follows, we develop mathematical models in more detail (see Table 1 for the details of notations and variables used in our models). Let each concentration of $U_i \ (i=1,2)$ in the membrane be given by $u_i(x,t)$, and that in the cytosol be given by $v_i(x, t)$, where $x\in [0, L]$ and $t\in [0, \infty )$. The general form of the membrane polarity model is given by

1 $$\begin{aligned}&\frac{\partial u_1}{\partial t}=D_{m_1}\frac{\partial ^2 }{\partial x^2} u_1+F_1(u_1, u_2, v_1, v_2) \nonumber \\&\frac{\partial v_1}{\partial t}=D_{c_1}\frac{\partial ^2 }{\partial x^2} v_1-F_1(u_1, u_2, v_1, v_2) \nonumber \\&\frac{\partial u_2}{\partial t}=D_{m_2}\frac{\partial ^2 }{\partial x^2} u_2+F_2(u_1, u_2, v_1, v_2)\nonumber \\&\frac{\partial v_2}{\partial t}=D_{c_2}\frac{\partial ^2 }{\partial x^2} v_2 -F_2(u_1, u_2, v_1, v_2) \end{aligned}$$

where $F_1(x, t)=\gamma v_1(x, t)-f_1(u_2(x, t))u_1(x, t)$, and $F_2(x, t)=\bar{\gamma } {v_2(x, t)} -f_2(u_1(x, t))u_2(x, t)$. These kinetic terms describe the transmembrane dynamics of two proteins, $U_1$ and $U_2$, where $\gamma (>0)$ and $\bar{\gamma }(>0)$ are constant on-rates, and $f_1$ and $f_2$ are off-rate functions describing a mutual inhibition effect with basal off-rates $\alpha (>0)$ and $\bar{\alpha }(>0)$. The detailed form of these functions are given by

$$\begin{aligned} f_1({u_2})=\alpha +\frac{K_1 u_2^2}{K+u_2^2}, \qquad f_2({u_1}) =\bar{\alpha }+\frac{\bar{K_1} u_1^2}{\bar{K}+u_1^2} \end{aligned}$$

where $K_1, \bar{K}_1, K$, and $\bar{K}$ are positive constants. Note that the details of kinetic terms may not be sensitive to our result as long as the kinetic terms satisfy the bi-stability property.

Next, we assume that the cytoplasmic protein *W* changes its type to slow or fast diffusive protein via membrane dependent phosphorylation by the protein, $U_1$ or $U_2$, and denote them by $W_s$ and $W_f$, respectively. We first assume the model of Type I, which has a slow diffusive phosphorylation cycle $W_s$ and is transited to a fast-diffusive type $W_f$ via $U_1$-dependent phosphorylation. This fast-diffusive type $W_f$ is transited to a slow diffusive type $W_s$ via $U_2$-dependent dephosphorylation. We also assume a contrasting phosphorylation cycle as the model of Type II where a fast diffusive type $W_f$ is transited to a slow diffusive type $W_s$ via $U_1$-dependent dephosphorylation and a slow diffusive type $W_s$ is transited to a fast diffusive type $W_f$ via $U_2$-dependent phosphorylation. The schematic diagrams are given in Fig. 1c. The Type I model is based on the MEX-5/6 dynamics and the Type II model is based on the PIE-1 dynamics. We give the details of both MEX-5/6 model and PIE-1 model in Sect. 3.3.

Now, let us define the concentrations of $W_s$ and $W_f$ by $w_s(x, t)$ and $w_f(x, t)$, respectively, and $w(x, t)=w_s(x, t)+w_f(x, t)$. Then, the general form of the cytosol polarity model describing the above dynamics can be considered as follows:

2 $$\begin{aligned}&\frac{\partial w_s}{\partial t}=D_{s}\frac{\partial ^2 }{\partial x^2} w_s +G(u_1, u_2, w_s, w_f), \nonumber \\&\frac{\partial w_f}{\partial t}=D_{f}\frac{\partial ^2 }{\partial x^2} w_f-G(u_1, u_2, w_s, w_f), \end{aligned}$$

where $D_f \ge D_s$. Type I and Type II dynamics define the function of *G*(*x*, *t*) as follows.

3 $$\begin{aligned}&\mathbf{Type }\ \mathbf{I: }\ W_s +U_1 \xrightarrow {~~\mu _1~~}W_f+U_1, \qquad W_f +U_2 \xrightarrow {~~\mu _2~~}W_s+U_2\nonumber \\&\mathbf{Type }\ \mathbf{II: }\ W_s +U_2 \xrightarrow {~~\mu _1~~}W_f+U_2, \qquad W_f +U_1 \xrightarrow {~~\mu _2~~}W_s+U_1 \end{aligned}$$

where $\mu _1(>0)$ is the conversion rate from the slow diffusive type to the fast diffusive type and $\mu _2(>0)$ is the conversion rate from the fast diffusive type to the slow diffusive type.

**Table 1:** Notations, variables, and parameters used in models

Protein name	Notation	Variable	Definition
Transmembrane protein 1	$U_1$	$u_1(x,t)$	Concentration in the membrane
		$v_1(x, t)$	Concentration in the cytosol
Transmembrane protein 2	$U_2$	$u_2(x,t)$	Concentration in the membrane
		$v_2(x, t)$	Concentration in the cytosol
Cytoplasmic protein	$W_s$	$w_s(x,t)$	Concentration of slow diffusive type
(*W*)	$W_f$	$w_f(x, t)$	Concentration of fast diffusive type

Notation	Parameter name	Notation	Parameter name
$\gamma , \bar{\gamma }$	On-rate	$K_1, \bar{K}_1$	Maximal off-rate
$\alpha , \bar{\alpha }$	Basal off-rate	$K, \bar{K}$	Degree of off-rate change
$\mu _1$	Conversion rate of $W_s\rightarrow W_f$	$\mu _2$	Conversion rate of $W_f\rightarrow W_s$

The chemical reaction formulas (3) directly lead to

4 $$\begin{aligned}&\mathbf{Type }\ \mathbf{I: }\ G(x, t)=\mu _2 u_2(x, t) w_f(x, t) -\mu _1 u_1(x, t) w_s(x, t), \nonumber \\&\mathbf{Type }\ \mathbf{II: }\ G(x, t)=\mu _2 u_1(x, t) w_f(x, t) -\mu _1 u_2(x, t) w_s(x, t). \end{aligned}$$

### Reduction to TFD and TSD models

**Fig. 2:**
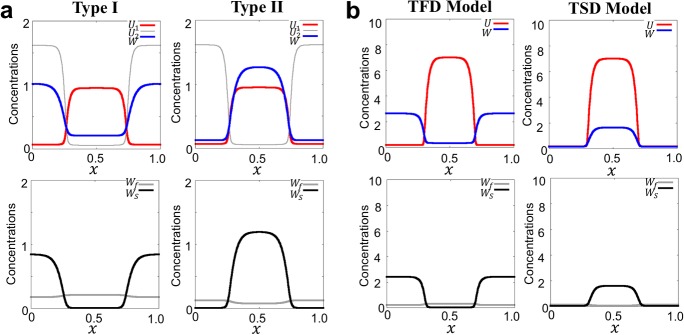
Representative simulation of in-phase and anti-phase polarities. **a** Results for Type I and Type II models. **b** Results for TFD and TSD models. Type I and TFD models show anti-phase polarity between *U* (red) and $W(=W_f+W_s)$ (blue), but Type II and TSD models show in-phase polarity. The second line panels show each concentration of slow diffusive type ($W_s$, black) and fast diffusive type ($W_f$, gray). The detailed parameter values and the initial conditions are given in “Appendix B” (color figure online)

To capture the essential structures of the cytoplasmic polarity mechanism, we reduce the upstream polarity model (1)–(4) to a more conceptional model that considers the core mathematical structure of polarity dynamics but is mathematically simpler. Let us reduce the membrane polarity model to the self-recruitment model (Fig. 1d) (see “Appendix A” for the details). The principal is that mutual inhibition can be considered to be equivalent to the dynamics of self-activation (Seirin-Lee and Shibata 2015). One can confirm that the dynamics of the reduction model are very similar to those of the full model (1) (Fig. 2). The reduction model for upstream and downstream polarities is given by

5 $$\begin{aligned}&\frac{\partial u}{\partial t}=D_{m}\frac{\partial ^2}{\partial x^2} u+F(u, v)\nonumber \\&\frac{\partial v}{\partial t}=D_{c}\frac{\partial ^2}{\partial x^2} v-F(u, v) \nonumber \\&\frac{\partial w_s}{\partial t}=D_{s}\frac{\partial ^2}{\partial x^2} w_s +G(u, w_s, w_f) \nonumber \\&\frac{\partial w_f}{\partial t}=D_{f}\frac{\partial ^2}{\partial x^2} w_f-G(u, w_s, w_f) \end{aligned}$$

where *u*(*x*, *t*) and *v*(*x*, *t*) are the membrane and cytosol concentrations of either $U_1$ or $U_2$ (in this paper, we consider it to be $U_1$). *F*(*x*, *t*) is given as

6 $$\begin{aligned} F(x, t)=\gamma v(x, t)-\left[ \alpha +\frac{\beta _2}{1+\beta _1 u(x, t)^2}\right] u(x, t), \end{aligned}$$

and the concentration of $U_2$ is reduced to

7 $$\begin{aligned} u_2(x, t)=\frac{\delta _2}{1+\delta _1 u(x, t)^2} \end{aligned}$$

where $\beta _1, \beta _2, \delta _1$, and $\delta _2$ are positive constants. Each definition of the constants is shown in “Appendix A”.

By substituting (7) into Type I and Type II of *G*(*x*, *t*), we obtain the transition to fast diffusive (TFD) and transition to slow diffusive (TSD) models as follows;

8 $$\begin{aligned}&\mathbf{TFD }\ \mathbf{Model: }\ G({x}, t)=\mu _2 \frac{\delta _2}{1+\delta _1 u({x}, t)^2} w_f({x}, t)-\mu _1 u({x}, t) w_s({x}, t), \end{aligned}$$

9 $$\begin{aligned}&\mathbf{TSD }\ \mathbf{Model: }\ G({x}, t)=\mu _2 u({x}, t) w_f({x}, t)-\mu _1 \frac{\delta _2}{1+\delta _1 u({x}, t)^2} w_s({x}, t), \end{aligned}$$

respectively. As shown in Fig. 1d, the TFD model describes that the upstream protein acts on a downstream protein to transit from the slow diffusive type to *fast diffusive type* (or *U* inhibits/activates the transition of slow/fast diffusive type of *W*). In contrast, the TSD model shows that the upstream protein acts on a downstream protein to transit from the fast-diffusive type to *slow diffusive type* (or *U* activates/inhibits the transition of slow/fast diffusive type of *W*).

## Results

### Transition to fast/slow diffusion induces anti-phase/in-phase cell polarity

We first highlight that the two conceptional models show two contrasting dynamics of patterning. Type I and TFD models show that *W* generates an anti-phase cytoplasmic polarity with respect to the membrane polarity of *U* (Fig. 2a, b left panels). In contrast, Type II and TSD models show that *W* generates an in-phase polarity with respect to the membrane polarity of *U* (Fig. 2a, b right panels). Furthermore, we found that in both models, the slow diffusive type $W_s$ and fast diffusive type $W_f$ also create different phases of polarity, and the polarity of $W_s$ coincides with the phase of *W*, indicating that $W_s$ strongly affects the total dynamics of *W* polarity.

From the model properties, we conclude that the anti-phase mechanism is based on the activation from slow type to fast type and the inhibition from fast type to slow type by an upstream polarity substrate. In contrast, the in-phase mechanism is based on the activation from fast type to slow type and the inhibition from slow type to fast type by an upstream polarity protein (see Fig. 5 and Sect. 4 for more details). These results suggest that the location of downstream polarity is dominated by the location of slow type polarity, and anti-/in-phase polarity in the cytosol is determined by regulating the upstream polarity protein.

**Fig. 3:**
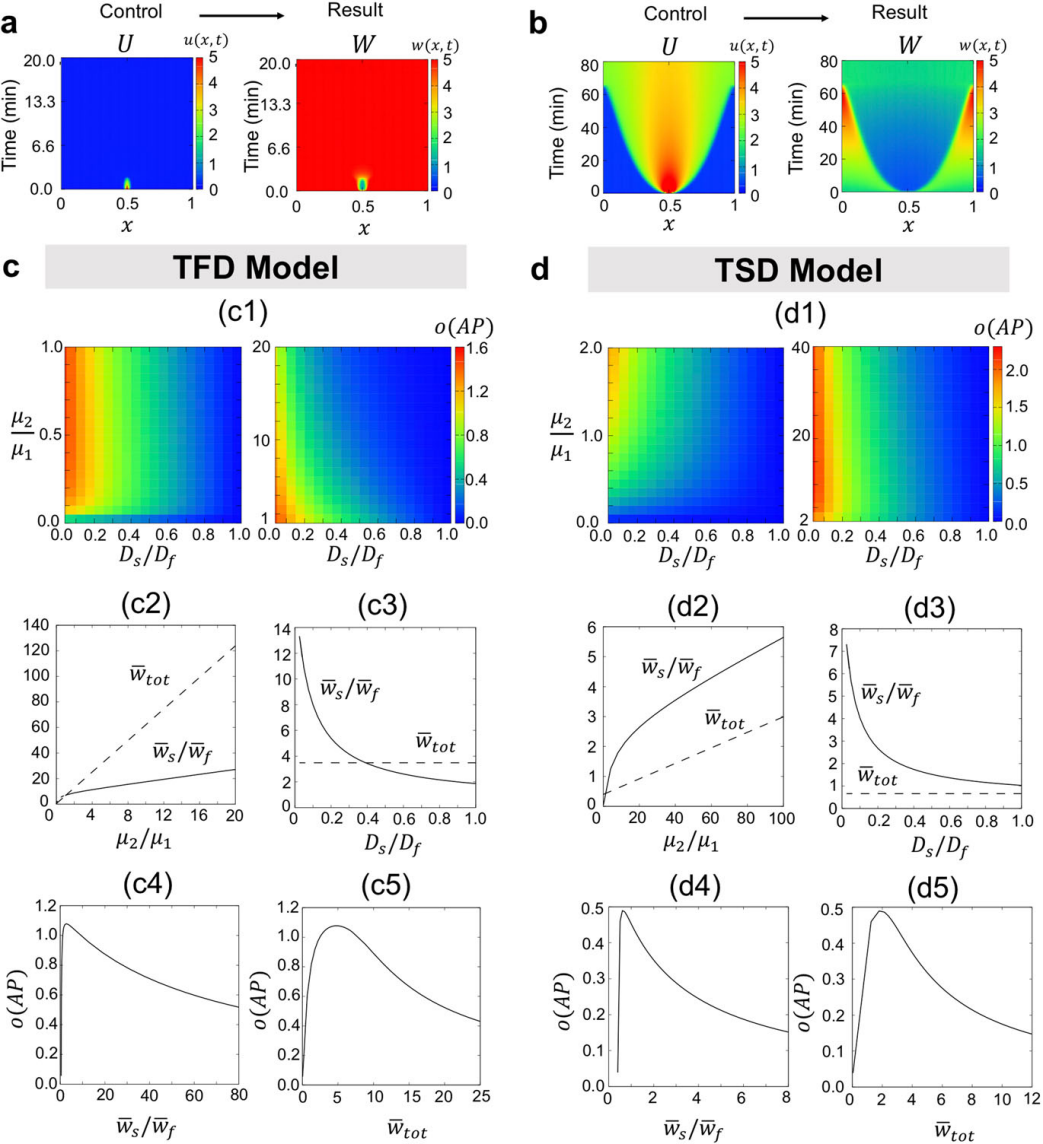
Underlying mechanism for downstream polarity formation. **a**, **b** Effect of upstream polarity of *U* on downstream polarity of *W* was simulated. The dynamics of *U* were controlled as failure cases of symmetry breaking (**a**) and maintenance phase (**b**). Under each condition, the result of *W* polarity is shown in the right side of figures. **c**, **d** Parameter space of TFD and TSD models for *W* polarity with respect to the ratio of conversion rates ($\mu _2/\mu _1$) and ratio of diffusion rates ($D_s/D_f$). The degree of apparent patterning (*o*(*AP*), Eq. 10) is plotted in c1 and d1. c2–c5 and d2–d5 show how the values of $\bar{w}_s/\bar{w}_f$ and $\bar{w}_{tot}$ change and are affected *o*(*AP*) in the simulation in c1 and d1. The detailed parameter values and the initial conditions are given in “Appendix B”

### Underlying mechanism for downstream polarity

To understand the core mechanism for downstream polarity formation, we investigated the effects of three factors that compose the downstream polarity model; (1) Existence of membrane protein(*U*) polarity, (2) proportion of diffusion rates ($D_s/D_f$), and (3) transit rates, $\mu _1$ (from the slow type to the fast type) and $\mu _2$ (from the fast type to slow type).

Firstly, we explored the existence of *W* polarity under the condition where *U* does not lead to symmetric breaking to see whether the symmetry breaking of *U* is indispensable to induce the symmetry breaking of *W* (Fig. 3a). Next, we investigated whether the polarity of *W* is maintained under the assumption that symmetry breaking is possible but the maintenance phase of *U* has failed (Fig. 3b). We found that both the symmetry breaking and the maintenance of polarity cannot be established without polarity formation of the membrane protein (*U*), indicating that both establishment and maintenance of membrane protein polarity are indispensable conditions for polarity formation of cytoplasmic protein *W*.

Next, under the condition where *U* creates a polarity, we explored how the diffusion and transit rates affect polarity formation of *W*. To evaluate whether downstream polarity is established, we defined the apparent patterning degree (*o*(*AP*)) such that

10 $$\begin{aligned} o(AP)=\frac{w_{\max }-w_{\min }}{\bar{w}_{tot}}, \end{aligned}$$

where $$w_{\max }=\max _{x\in [0,L]}w(x, \infty )$$, $$w_{\min }=\min _{x\in [0,L]}w(x, \infty )$$, and

$$\begin{aligned} \bar{w}_{tot}=\frac{1}{L}\int _0^L w(x, \infty )dx. \end{aligned}$$

Note that $\bar{w}_{tot}$ denotes both the total concentration of *W* and the concentration of homogeneous steady state of *W* in scaled space [0, 1]. Thus, $o(AP)=0$ implies that $w_{\max }=w_{\min }=\bar{w}_{tot}$. The higher value of *o*(*AP*) indicates that the polarity in the cytosol is more apparent.

We first investigated the parameter space of $D_s/D_f$ and $\mu _2/\mu _1$ with respect to *o*(*AP*) in Fig. 3c, d. We found that both TFD and TSD models show very similar results, indicating that these four parameters do not essentially influence the location of polarities (either anti- or in-phase polarity exists), but they are commonly related with the existence of cytoplasmic polarity. The simulation results show that polarity exists in a proper range of $\mu _2/\mu _1$, and patterning is not apparent when $\mu _2/\mu _1$ is either very small or large. In contrast, patterning monotonically increases as $D_s/D_f$ decreases. We found that when the diffusion rates differ slightly, the concentration *W* is in a homogeneous state, although $W_s$ and $W_f$ generate clear polarities (Fig. 6 in “Appendix”). This suggests that the symmetry breaking of *W* is dominated by not only the asymmetry of the two transition rates but also the perturbation by different diffusion rates, which are indispensable.

Next, we investigate the reason of the influence of transition and diffusion rates on *o*(*AP*). Because the model is conservative, we first focused on the total mass of *W* ($\bar{w}_{tot}$) and the proportion of total mass of $W_s$ and $W_f$, denoted by $\bar{w}_{s}$ and $\bar{w}_{f}$, respectively (Fig. 3c2–c5, d2–d5), where

$$\begin{aligned} \bar{w}_{s}=(1/L)\int _0^L w_s(x, \infty ) dx ~~\text {and}~~ \bar{w}_{f}=(1/L)\int _0^L w_f(x, \infty ) dx. \end{aligned}$$

We found that both $\bar{w}_{tot}$ and $\bar{w}_s/\bar{w}_f$ are proportional to $\mu _2/\mu _1$, and $\bar{w}_s/\bar{w}_f$ is also proportional to $D_s/D_f$ (Fig. 3c2–c3, d2–d3). We confirmed *o*(*AP*) with respect to $\bar{w}_{tot}$ and $\bar{w}_s/\bar{w}_f$ and found that the polarity cannot be formed when $\bar{w}_{tot}$ and $\bar{w}_s/\bar{w}_f$ are either very small or large (Fig. 3c4–c5, d4–d5 ). This result shows that the concentration valance of slow and fast types and the total mass are important to determine the degree and shape of polarity. In contrast, we found that there are no notable differences between the TFD and TSD models with respect to the above results. This indicates that the mathematical structure for forming the polarity is the same between the two models, and the location of polarity is purely determined by the properties of model formulation.

### Dynamics of up–mid–down streams polarity in *C. elegans* embryo; PARs, MEX-5/6, and PIE-1 models

Here, we show that the multiple streams polarity dynamics of PARs, MEX-5/6, and PIE-1 in *C. elegans* embryo can be understood by our conceptional models. For this purpose, we first construct the phenomenological model of PARs, MEX-5/6, and PIE-1 based on biological data.

The MEX-5/6 model including PARs dynamics is considered to be the Type I model in our study. It has been found that MEX-5/6 exists as two diffusive types of slow and fast diffusions in the cytosol, and the conversion of diffusion type is regulated by phosphorylation and dephosphorylation via pPAR and aPAR dependent manners (Daniels et al. 2010; Wu et al. 2018). It has been suggested that the slow diffusive type of MEX-5/6 is converted to a fast diffusive type via pPAR-dependent phosphorylation, and a fast diffusive type of MEX-5/6 is converted to a slow diffusive type via aPAR-dependent dephosphorylation (Daniels et al. 2010; Hoege and Hyman 2013). The conceptional diagram for MEX-5/6 dynamics can be described as that shown in Fig. 1c (Type I) by replacing $U_1\equiv$ pPARs with $U_2\equiv$ aPARs. Thus, we can directly reconsider the MEX-5/6 model as the TFD model. By replacing *u*(*x*, *t*) and *v*(*x*, *t*) with $[P_m](x,t)$ and $[P_c](x,t)$, which are the concentrations of posterior PAR proteins in the membrane and cytosol, respectively, and $w_s(x,t)$ and $w_f(x,t)$ with $[M_s](x,t)$ and $[M_f](x,t)$, which are the concentrations of MEX-5/6 of slow and fast diffusive types, respectively, in the model (5) of TFD, we can directly formulate the PARs-MEX-5/6 model as follows.

11 $$\begin{aligned}&\frac{\partial [P_m]}{\partial t}=D_{m}\frac{\partial ^2}{\partial x^2} [P_m] +\gamma [P_c] -\left\{ \alpha +\frac{\beta _2}{1+\beta _1 [P_m]^2}\right\} [P_m],\nonumber \\&\frac{\partial [P_c]}{\partial t}=D_{c}\frac{\partial ^2}{\partial x^2} [P_c] -\gamma [P_c]+\left\{ \alpha +\frac{\beta _2}{1+\beta _1 [P_m]^2}\right\} [P_m], \end{aligned}$$

12 $$\begin{aligned}&\frac{\partial [M_s]}{\partial t}=D_{s}\frac{\partial ^2}{\partial x^2} [M_s] +\mu _2 \frac{\delta _2}{1+\delta _1 [P_m]^2} [M_f]-\mu _1 [P_m] [M_s],\nonumber \\&\frac{\partial [M_f]}{\partial t}=D_{f}\frac{\partial ^2}{\partial x^2} [M_f] -\mu _2 \frac{\delta _2}{1+\delta _1 [P_m]^2} [M_f]+\mu _1 [P_m] [M_s]. \end{aligned}$$

We define $$[M](x,t)=[M_s](x,t)+[M_f](x,t)$$.

**Fig. 4:**
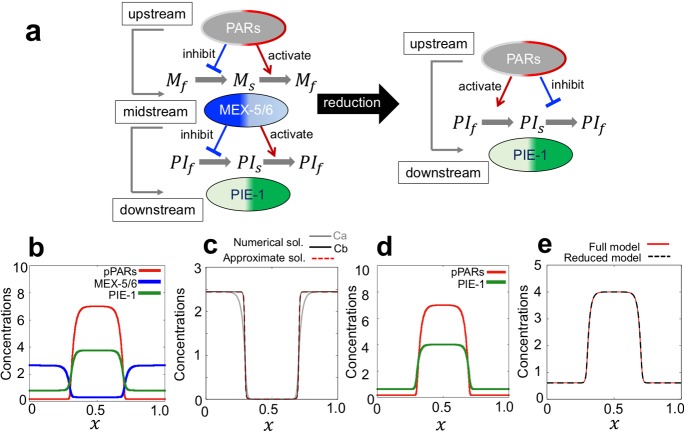
Schematic diagram for model reduction and simulation results for PIE-1 polarity formation. **a** Schematic diagram of PARs-PIE-1 Model. **b** Representative simulation results of the PARs-MEX-5/6-PIE-1 model (11)–(13). **c** The comparison of approximated solution (dotted line) of $[M_s]$ given by (16), and the numerical solutions (dashed line) of $[M_s]$ from the PARs-MEX-5/6-PIE-1 model (11)–(13) in sufficiently large time. Ca is the case for $D_s=1.28\times 10^{-4}$, which is the same as the simulation results (**b**). Cb is the case for $D_s=1.28\times 10^{-6}$. **d** Representative simulation result of the PIE-1 reduced model (17). The result shows that PIE-1 shows *in-phase* polarity with respect to PAR polarity. **e** The comparison of PIE-1 solutions solved by the PARs-MEX-5/6-PIE-1 full model (11)–(13) with $D_s=1.28\times 10^{-6}$ (red line) and PIE-1 reduced model (17) (dotted black line) in sufficiently large time. The detailed parameter values and the initial conditions are given in “Appendix B” (color figure online)

Next, we extend the PARs-MEX-5/6 model (11)–(12) with PIE-1 dynamics. PIE-1 is a cytoplasmic protein generating a polarity in the opposite side of MEX-5/6 polarity. Thus, PIE-1 creates in-phase polarity to posterior PAR polarity in contrast to MEX-5/6 which creates anti-phase polarity to posterior PAR polarity (Fig. 1a). PIE-1 is downstream of MEX-5/6, and thus PAR polarity, MEX-5/6 polarity, and PIE-1 polarity are related by upstream, midstream, and downstream dynamics (Fig. 4a). The underlying mechanism of PIE-1 forming a polarity is known to be based on conversion of diffusion type via a phosphorylation cycle regulated by MEX-5/6 concentration (Daniels et al. 2009; Wu et al. 2015, 2018). Wu et al. (2015, 2018) proposed that MEX-5/6 regulates the distribution of PIE-1 slow type particles, and PIE-1 mobility is proportional to MEX-5/6 concentration. Thus, we assume that the transit rate from the slow diffusive type to the fast-diffusive type of PIE-1 is proportional to MEX-5 concentration such that

$$\begin{aligned} \text {Transition rate from slow to fast type }\equiv \gamma _1 [M](x,t) \end{aligned}$$

where $\gamma _1$ is a positive constant determining the transit rate. In contrast, Wu et al. (2018) found that the transition rate from the fast diffusive type to the slow diffusive type of PIE-1 increases from the anterior side (the side of high concentration of MEX-5/6) to the posterior side (the side of low concentration of MEX-5/6), and it becomes almost uniform in the spatially homogeneous state of MEX-5/6 concentration. Thus, we assume that the transition rate from the fast diffusive type to the slow diffusive type of PIE-1 decreases when MEX-5/6 increases and it becomes constant when MEX-5/6 is absent such that

$$\begin{aligned} \text {Transit rate from fast to slow type } \equiv \frac{\gamma _2}{ 1+\gamma _3[M](x,t)} \end{aligned}$$

where $\gamma _2$ is the basal transition rate of PIE-1 from the fast to slow type, and $\gamma _3$ is the inhibition rate by MEX-5/6. Hence, the two assumptions above lead to the following PIE-1 model.

13 $$\begin{aligned}&\frac{\partial [PI_s]}{\partial t}=D_{ps}\frac{\partial ^2}{\partial x^2} [PI_s] +\frac{\gamma _2}{ 1+\gamma _3[M]}[PI_f]-\gamma _1[M][PI_s], \nonumber \\&\frac{\partial [PI_f]}{\partial t}=D_{pf}\frac{\partial ^2}{\partial x^2} [PI_f] -\frac{\gamma _2}{ 1+\gamma _3[M]}[PI_f]+\gamma _1[M][PI_s], \end{aligned}$$

where $[PI_s](x,t)$ and $[PI_f](x,t)$ are the concentrations of the slow and fast diffusive types of PIE-1.

We first found that the model of PARs, MEX-5/6, and PIE-1 (11)–(13) successfully forms anti-phase PIE-1 polarity with respect to the polarity peak of MEX-5/6 in the cytosol, which results in an in-phase relation with the polarity of PARs of the membrane (Fig. 4b).

Next, we investigate that the dynamics of PIE-1 can be considered as a type of TSD model with respect to PAR polarity via heuristic approximation. We show that PIE-1 dynamics can essentially be reconsidered as direct downstream dynamics of PARs (Fig. 4a). The numerical results of Fig. 2 show that stationary solutions exist for the system (11)–(12). Let us consider the stationary problem of the model (12) for sufficiently small $D_s$ (i.e. $D_s\rightarrow 0$) such that

14 $$\begin{aligned}&0=\mu _2 \frac{\delta _2}{1+\delta _1 [\overline{P_m}]^2} [\overline{M_f}] -\mu _1 [\overline{P_m}] [\overline{M_s}],\nonumber \\&0=D_{f}\frac{\partial ^2}{\partial x^2} [\overline{M_f}]-\mu _2 \frac{\delta _2}{1+\delta _1 [P_m]^2} [\overline{M_f}] +\mu _1 [\overline{P_m}] [\overline{M_s}], \end{aligned}$$

where $[\overline{P_m}]$, $[\overline{M_s}]$, and $[\overline{M_f}]$ are stationary solutions of the model (11). From Eq. (14), we obtain

$$\begin{aligned} D_{f}\frac{\partial ^2}{\partial x^2} [\overline{M_f}]=0 \end{aligned}$$

with respect to the periodic boundary conditions. Thus, $[\overline{M_f}]$ should be a constant stationary solution. Let us denote it by ${M}_f^*$. Then, from (14), we have

15 $$\begin{aligned} {[}\overline{M_s}](x) =\frac{\mu _2 \delta _2 M_f^*}{\mu _1 [\overline{P_m}](x) \{1+\delta _1 [\overline{P_m}](x)^2\}}. \end{aligned}$$

Based on (15), we approximate the solution of the slow diffusive type of MEX-5/6 on $[0, L]\times (0,~\infty )$ to

16 $$\begin{aligned} {[}M_s](x,t) \approx \frac{\mu _2 \delta _2 M_f^*}{\mu _1 [P_m](x,t) \{1+\delta _1 [P_m](x,t)^2\}}. \end{aligned}$$

Note that the approximation solution given by (16) coincides well with the original solution solved by (12) with respect to small $D_s$ (Fig. 4c).

Now, let us substitute Eq. (16) into the PIE-1 model (13). We obtain the PIE-1 model including the direct interaction with PARs as follows.

17 $$\begin{aligned}&\frac{\partial [PI_s]}{\partial t}=D_{ps}\frac{\partial ^2}{\partial x^2} [PI_s] +p([P_m])[PI_f]-q([P_m])[PI_s], \nonumber \\&\frac{\partial [PI_f]}{\partial t}=D_{pf}\frac{\partial ^2}{\partial x^2} [PI_f] -p([P_m])[PI_f]+q([P_m])[PI_s]. \end{aligned}$$

*p*(*u*) and *q*(*u*) are given by

$$\begin{aligned} p(u)=\frac{{A_2}(A_1 u+ u^3)}{A_0+A_1 u+u^3}, \quad q(u)=B_0+\frac{B_3}{B_1 u+B_2 u^3} \end{aligned}$$

where $$A_0=\gamma _3 \mu _2 \delta _2 M_f^*/(\mu _1\delta _1+\mu _1\delta _1\gamma _3 M_f^*), ~A_1=1/\delta _1, ~A_2=\gamma _2/(1+\gamma _3 M_f^*), ~B_0=\gamma _1M_f^*, ~B_1=\mu _1, B_2=\mu _1\delta _1,$$ and $$B_3=\gamma _1\mu _2\delta _2 M_f^*$$.

Note that the model (17) is independent of MEX-5/6 and is directly described by PARs. Furthermore, we can simply show that *p*(*u*) is a monotonically increasing function of $u(>0)$ for arbitrary positive parameters (check $p'(u)>0$). This implies that the upstream protein PAR activates the transiting effect of diffusive type of PIE-1 from fast to slow. In contrast, we can directly show that *q*(*u*) is a monotonically decreasing function of $u(>0)$ for arbitrary positive parameters. This indicates that the upstream protein PAR inhibits the transiting effect of diffusive type of PIE-1 from slow to fast. That is, the model formulation (17) can be described as a type of TSD model. We confirm the model (17) with (11) in a numerical simulation, and Fig. 4d shows that PIE-1 forms in-phase polarity with respect to PAR polarity. We also confirmed that the PIE-1 solution of the reduced PIE-1 model (17) coincides with that of the full model (11)–(13) (Fig. 4e).

## Mathematical analysis

### Mathematically general property for in- and anti-phase of polarity in a cell

**Fig. 5:**
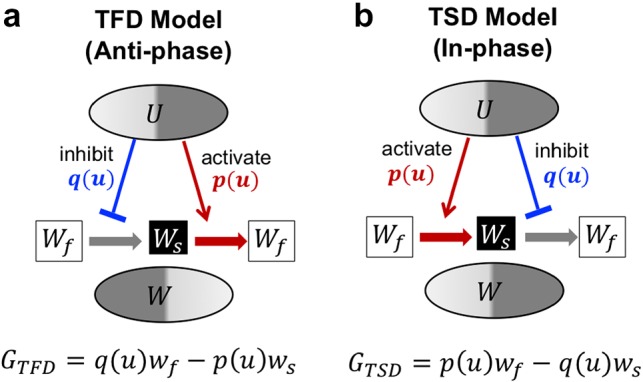
General properties for the anti-phase TFD model and the in-phase TSD model. **a** and **b** Show a general condition for anti-phase and in-phase patterning, respectively. *p*(*u*) is a monotonically increasing function and *q*(*u*) is a monotonically decreasing function

Considering the result of approximating MEX-5/6 model and PIE-1 model, we hypothesize that TFD and TSD models can be extended to a general type model without dependency of specific kinetic forms. From the simulation observations in the previous section, we found that the positioning (either *anti-phase* or *in-phase*) of downstream polarity to the upstream polarity is determined by either the transition of [Slow type $\rightarrow$ Fast type] is *activated* and [Fast type $\rightarrow$ Slow type] is *inhibited* by the upstream polarity protein (Fig. 5a), or the transition of [Fast type $\rightarrow$ Slow type] is *activated* and [Slow type $\rightarrow$ Fast type] is *inhibited* by the upstream polarity protein (Fig. 5b). In what follows, we show mathematically general property for *anti-phase* and *in-phase* with respect to a monotone increasing function *p*(*u*) (the activation effect by upstream polarity substrate) and a monotone decreasing function *q*(*u*) (the inhibition effect by upstream polarity substrate).

A general type model describing these properties can be given by

18 $$\begin{aligned} \left\{ \begin{array}{l} \dfrac{\partial u}{\partial t}=d_1\dfrac{\partial ^2 u}{\partial x^2} + f(u,v),\\ \dfrac{\partial v}{\partial t} =d_2\dfrac{\partial ^2 v}{\partial x^2} - f(u,v),\\ \dfrac{\partial w_s}{\partial t}=d_{s}\dfrac{\partial ^2 w_s}{\partial x^2}+ g(u,w_s,w_f),\\ \dfrac{\partial w_f}{\partial t}=d_{f} \dfrac{\partial ^2w_f}{\partial x^2}-g(u,w_s,w_f), \end{array}\right. \qquad x \in I, \quad t>0. \end{aligned}$$

where *u*(*x*, *t*), *v*(*x*, *t*) are concentrations of upstream proteins, and $w_s(x,t), w_f(x,t)$ are concentrations of downstream proteins. $d_1, d_2, d_s, d_f$ represent diffusion coefficients of each protein and we suppose $0<d_1\le d_2$ and $0<d_s \le d_f$. Let *f* and *g* denote reaction terms for upstream proteins and downstream proteins, and we consider *f* and *g* are smooth functions. Applying variable conversion, we can put $I:=[-L/2,L/2]$. This formulation makes our mathematical formulation be simpler, and the mathematical results hold without loss of generality, because we consider periodic boundary condition for (18).

If $(u,v,w_s,w_f)$ is solution to (18), it has conserved quantities independent of variable *t*, given by

19 $$\begin{aligned} \theta&:= \dfrac{1}{L}\int _I(u(x,t)+v(x,t))dx\equiv \dfrac{1}{L} \int _I(u(x,0)+v(x,0))dx,\nonumber \\ \xi&:=\dfrac{1}{L}\int _I(w_s(x,t)+w_f(x,t))dx \equiv \dfrac{1}{L}\int _I(w_s(x,0)+w_f(x,0))dx, \end{aligned}$$

The quantities $\theta$ and $\xi$ are the spatial averages of the upstream proteins and the downstream proteins, respectively. We can prove the first equation of (19) by applying differentiation under the integral sign and periodic boundary condition such that

$$\begin{aligned} L\dfrac{d \theta }{dt}&=\dfrac{d}{dt}\int _I(u(x,t)+v(x,t))dx\\&=\int _I\left( \dfrac{\partial u}{\partial t}(x,t) +\dfrac{\partial v}{\partial t}(x,t)\right) dx =\int _I\left( d_1\dfrac{\partial ^2 u}{\partial x^2}(x,t) +d_2\dfrac{\partial ^2 v}{\partial x^2}(x,t)\right) dx\\&=\left[ d_1\dfrac{\partial u}{\partial x}(x,t) +d_2\dfrac{\partial v}{\partial x}(x,t)\right] _{x=-L/2}^{x=L/2}=0. \end{aligned}$$

Thus, $\theta$ is constant. Similarly, we can prove the second equation of (19).

Note that the spatial averages are not unique pair of conserved quantities for Eq. (18) because $c_1 \theta$ and $c_2 \xi$ are also conserved quantities of (18), where $c_1$ and $c_2$ are arbitrary real numbers. Thus, we define the conserved quantity as a spatial average, without loss of generality in order for the mathematical analysis to be simpler.

In this section, we consider stationary solutions of the system (18) when (*u*(*x*, *t*), *v*(*x*, *t*)) converges to a stable non-constant stationary solution $(u^*(x),v^*(x))$; it corresponds to the condition that upstream proteins form a stable polarity pattern. In this case, Eq. (18) is transformed to

$$\begin{aligned} \left\{ \begin{array}{l} 0=d_1\dfrac{d^2 u^*}{d x^2} + f(u^*,v^*),\\ 0=d_2\dfrac{d^2 v^*}{d x^2} - f(u^*,v^*),\\ \dfrac{\partial w_s}{\partial t}=d_s\dfrac{\partial ^2 w_s}{\partial x^2} +g(u^*,w_s,w_f),\\ \dfrac{\partial w_f}{\partial t}=d_f \dfrac{\partial ^2w_f}{\partial x^2}-g(u^*,w_s,w_f), \end{array}\right. \qquad x \in I,\quad t > 0. \end{aligned}$$

We apply variable conversion $x \rightarrow x/\sqrt{d_f}$, then time evolution equation of $w_s, w_f$ is given by

20 $$\begin{aligned}&\left\{ \begin{array}{l} \dfrac{\partial w_s}{\partial t} =D\dfrac{\partial ^2 w_s}{\partial x^2} +g^*(w_s,w_f)\\ \dfrac{\partial w_f}{\partial t} =\dfrac{\partial ^2w_f}{\partial x^2}-g^*(w_s,w_f), \end{array}\right. \qquad x \in I, \quad t > 0.\nonumber \\&g^*(x,t):=g(u^*(x),w_s(x,t),w_f(x,t)). \end{aligned}$$

Now, we set $D:=d_s/d_f$, and replace $L/\sqrt{d_f}$ by *L*. Suppose that $(w_s^*, w_f^*)$ be a stationary solution of Eq. (20). Then, it satisfies

21 $$\begin{aligned} \left\{ \begin{array}{l} D\dfrac{d^2 w_s^*}{dx^2} +g^*(w_s^*,w_f^*)=0,\\ \dfrac{d^2w_f^*}{dx^2}-g^*(w_s^*,w_f^*)=0,\\ \langle w_s^* \rangle + \langle w_f^* \rangle =\xi , \end{array}\right. \qquad x \in I, \quad t > 0. \end{aligned}$$

The third equation denotes a conserved quantity. $\langle \phi \rangle$ means the spatial average, where

$$\begin{aligned} \langle \phi \rangle := (1/L)\int _I\phi (x)dx. \end{aligned}$$

Our main mathematical result is the shape of stable non-constant solution of the system (21) and this result proposes that the polarity of downstream proteins is formed when the polarity of the upstream proteins is formed. Note that if $(u^*,~v^*)$ is unstable, then stationary solution $(u^*, v^*, w_s^*, w_f^*)$ for the system (18) becomes unstable. This is because the equation of *u* and *v* are independent of $w_s$ and $w_f$. Therefore, we here assume the existence and stability of non-constant stationary state $(u^*(x),v^*(x))$.

In what follows, we first introduce basic assumptions in the Sect. 4.2, and then explain the main results and proofs in the Sects. 4.3 and 4.4.

### Basic assumptions

Let us first consider the system of only *u* and *v* as the following.

22 $$\begin{aligned} \left\{ \begin{array}{l} \dfrac{\partial u}{\partial t} =d_1\dfrac{\partial ^2 u}{\partial x^2} + f(u,v),\\ \dfrac{\partial v}{\partial t} =d_2\dfrac{\partial ^2 v}{\partial x^2} - f(u,v). \end{array}\right. \quad x \in I, \quad t > 0. \end{aligned}$$

#### Assumption 1

Equation (22) has a stationary solution $S(x)=(u^*(x),v^*(x))$, where $u^*, v^*$ are smooth functions in I, and satisfy

(i) $$u^{*}(x)>0, v^{*}(x) > 0 \quad (x \in I)$$
(ii) $u^{*}$ and $v^{*}$ are strictly decreasing and increasing, respectively, in *x* for $0<x<L/2$.
(iii) $u^{*}$ and $v^{*}$ are even periodic functions with period *L*.

First condition is very obvious, because $u^*$ and $v^*$ are concentrations of chemical substrates. Second condition assumes the shape of upstream polarity. Equation (22) is invariant under the transformation $x \rightarrow -x$, thus we assume the condition (iii). We regard $\theta$ as a parameter of solution, and denote $S(x)=S(x;\theta )$.

Next, we assume the stability of *S*(*x*). Let us denote ${\mathcal {L}},~dom({\mathcal {L}})$, and $\sigma ({\mathcal {L}})$ as linearized operators of the right hand side equations in (22) at *S*(*x*), domain of ${\mathcal {L}}$, and spectral set of ${\mathcal {L}}$.

$$\begin{aligned} dom({\mathcal {L}})&:= X=\left\{ \varvec{v}=^{T}(v_1,v_2) \in H^2_p(I) \times H^2_p(I) ;\ \int _I(v_1+v_2)dx = 0\right\} , \\ H^2_p(I)&:= \left\{ u \in H^2(I) ; \ u(-L/2)=u(L/2), \quad u_x(-L/2)=u_x(L/2) \right\} , \\ {\mathcal {L}} \varvec{v}&:=\begin{pmatrix} d_1 &{} 0 \\ 0 &{} d_2 \end{pmatrix} \partial ^2_x\varvec{v} + \begin{pmatrix} f^*_u &{} {f}^*_v \\ -f^*_u &{} -{f}^*_v \end{pmatrix} \varvec{v}, \quad \text {where}\quad {f}^*_u := f_u(u^*,v^*), \quad {f}^*_v:=f_v(u^*,v^*). \end{aligned}$$

From Assumption 1, *S*(*x*) is even periodic function on *I*. Then its derivative $S_x(x)=(u^*_x(x),v^*_x(x))$ is odd periodic function on *I*. Accordingly, $\int _{I}(u^*_{x}+v^*_{x})dx=0$. Moreover, substituting *S*(*x*) into the Eq. (22) and differentiating the equation by *x*, we obtain ${\mathcal {L}}S_x=0$. Therefore, 0 is eigenvalue of ${\mathcal {L}}$, and $S_x$ is the corresponding eigenfunction. Note that $S_{\theta }$ also satisfies ${\mathcal {L}}S_{\theta }=0$. However, $S_{\theta } \notin dom({\mathcal {L}})$, because $\int _{I}(u^*_{\theta }+v^*_{\theta })dx=L$. Therefore, $S_{\theta }$ is not eigenfunction of ${\mathcal {L}}$.

#### Assumption 2

(i) 0 is simple eigenvalue of ${\mathcal {L}}$ with the eigenfunction $S_x$.
(ii) There exists some $\delta >0$, such that $\sigma ({\mathcal {L}}) \subset \{ \lambda \in {\mathbb {C}} ; Re(\lambda ) < -\delta \} \cup \{ 0 \}$, where $Re(\lambda )$ denotes real part of $\lambda$.

We only consider the case that upstream proteins generate a stable polarity, hence we assume the condition above. Mathematically, this assumption is sufficient condition that *S*(*x*) is stable except the translation.

Finally, we set the assumption for the mathematical condition of *g*.

#### Assumption 3

Let *p* and *q* be smooth functions, and suppose that *p* and *q* are strictly increasing and decreasing functions in ${\mathbb {R}}$. Moreover, we assume $p(u)> 0, q(u)> 0 \quad (u > 0)$. We suppose *g* satisfies the condition (*TFD*) or (*TSD*).

$$\begin{aligned}&(TFD) \quad g(x,t)=q(u(x,t))w_f(x,t) - p(u(x,t))w_s(x,t).\\&(TSD) \quad g(x,t)=p(u(x,t))w_f(x,t) - q(u(x,t))w_s(x,t). \end{aligned}$$

(*TFD*) and (*TSD*) are corresponding to the general conditions for *in-phase* and *anti-phase* polarity.

In next subsection, we prove the existence of non-constant stationary solution of the system (20) under Assumptions 1 and 3. In what follows, we explain all proof under the case of (*TFD*) for simplicity and only remark the results for the case of (*TSD*), because we can prove the results for (*TSD*) by the same manner with (*TFD*).

### The existence of non-constant stationary solutions

For the case of (*TFD*), the system (21) is given by

23 $$\begin{aligned} \left\{ \begin{array}{l} D\dfrac{d^2 w_s}{dx^2} - p^*(x)w_s + q^*(x)w_f=0,\\ \dfrac{d^2 w_f}{dx^2} + p^*(x)w_s - q^*(x)w_f=0,\\ \langle w_s \rangle + \langle w_f \rangle =\xi , \end{array}\right. \qquad x \in I. \end{aligned}$$

We consider the solution of the system (23) by $(w_s(x),w_f(x)){=}(w_s(x;\xi ), w_f(x;\xi ))$. Note that the general solution is represented by $(w_s(x;\xi ), w_f(x;\xi ))=\xi (w_s(x;1), w_f(x;1))$ if there exist a solution $(w_s(x;1), w_f(x;1))$ for $\xi =1$, because every term in the left hand side of Eq. (23) is linear about $w_s$ and $w_f$. Therefore, we only need to consider the case of $\xi =1$.

Taking the sum of first and second equations in the system (23), we obtain

$$\begin{aligned} D\dfrac{d^2 w_s}{d x^2} + \dfrac{d^2 w_f}{d x^2} = 0, \qquad x \in I. \end{aligned}$$

Applying the indefinite integration with periodic boundary conditions, we obtain

24 $$\begin{aligned} D w_s(x;1) + w_f(x;1) = C, \qquad x \in I, \end{aligned}$$

where *C* is an integration constant. Taking the spatial average of the equation above, we obtain

$$\begin{aligned} C&= D \langle w_s \rangle + \langle w_f \rangle ,\\&=D(1 - \langle w_f \rangle ) + \langle w_f \rangle , \\&=D + (1 - D)\langle w_f \rangle , \end{aligned}$$

because $\langle w_s \rangle + \langle w_f \rangle =1$. Substituting *C* into (24), we obtain

$$\begin{aligned} {w}_s(x;1) = 1 + \dfrac{1-D}{D}\langle {w}_f \rangle - \dfrac{1}{D} w_f(x;1). \end{aligned}$$

Substituting $w_s$ into the second equation of (23), we finally obtain the equation containing $w_f$ only as the following.

25 $$\begin{aligned} \left\{ \begin{array}{l} \dfrac{d^2 {w}_f}{d x^2} - \rho (x){w}_f + \dfrac{1-D}{D} \langle {w}_f \rangle p^*(x)= -p^*(x),\\ {w}_s(x;1) = 1 + \dfrac{1-D}{D}\langle {w}_f \rangle - \dfrac{1}{D}{w}_f(x;1), \end{array}\right. \qquad x \in I, \end{aligned}$$

where $$\rho (x):=D^{-1}p^*(x) + q^*(x)\ (0<D\le 1)$$.

#### Theorem 4.1

Suppose Assumption 1 and (*TFD*) hold, then (25) has unique solution $(w_{f}^{*}, w_{s}^{*})$ satisfying the following (i)–(iii)

(i) $$w^*_f(x)> 0,\quad w_{s}^{*}(x) > 0 \qquad (x \in I)$$.
(ii) $w_f^*$ and $w_s^*$ are even and periodic functions with period *L*.
(iii) $w_{f}^{*}$ and $w_{s}^{*}$ are strictly decreasing and increasing functions for $x \in (0,L/2)$.
  Moreover, we denote $w^*(x):=w^*_f(x)+w^*_s(x)$ and suppose $D\ne 1$, then $w^*$ satisfies (i) and (ii), and $w^*$ is strictly increasing function for $x \in (0,L/2)$.

#### Remark 4.1

If we replace the condition (*TFD*) with (*TSD*) in Theorem 4.1, $w_{f}^{*}$ and $w_{s}^{*}$ are strictly increasing and decreasing in (0, *L*/2). Moreover, $w^*$ is strictly decreasing in (0, *L*/2), for $D\ne 1$.

Theorem 4.1(iii) proposes that the case of (*TFD*) makes anti-phase polarity and Remark 4.1 proposes that the case of (*TSD*) makes in-phase polarity.

#### Remark 4.2

From Theorem 4.1, (23) has a unique solution for any $\xi \in {\mathbb {R}}$, and the solution is represented by $(w_s(x;\xi ), w_f(x;\xi )) = \xi (w^*_s(x), w^*_f(x))$. Therefore, if $\xi >0$, then $w_s$ and $w_f$ also satisfy (i)$\sim$(iii).

First equation of (25) is linear integro-differential equation. One of the useful tools for solving this type equation is *Sherman–Morrison–Woodbury’s formula* (Wu 2016), and we can find a unique solution represented by

$$\begin{aligned} w_{f}^{*}(x)=({\mathcal {H}}+{\mathcal {Q}})^{-1} \left( -p^*(x)\right) =\left( {\mathcal {H}}^{-1} - \frac{1}{c_f}{\mathcal {H}}^{-1}{\mathcal {Q}} {\mathcal {H}}^{-1}\right) \left( -p^*(x)\right) , \end{aligned}$$

where

$$\begin{aligned} {\mathcal {H}}\phi :=\dfrac{d^2\phi }{dx^2}-\rho (x)\phi , \quad {\mathcal {Q}} u :=\dfrac{1-D}{D} \langle u \rangle p^*(x), \quad c_f:= 1 + \dfrac{1 - D}{D}\langle {\mathcal {H}}^{-1} p^{*} \rangle ,\nonumber \end{aligned}$$

if $c_f\ne 0$ and the inverse operator ${\mathcal {H}}^{-1}$ exists.

To justify above, we consider following equations under periodic boundary condition.

26 $$\begin{aligned} \dfrac{d^2 k}{d x^2} - \rho (x)k = -p^*(x), \quad x \in I. \end{aligned}$$

#### Lemma 4.1

Suppose Assumption 1 and (TFD) or (TSD) hold, then Eq. (26) has a unique solution under the periodic boundary condition.

#### Lemma 4.2

Suppose Assumption 1 and (TFD) hold, and let $k^{*}$ be a solution of Eq. (26) under periodic boundary condition, then $k^{*}$ satisfies

(i) $k^{*}$ is even periodic function with period *L*,
(ii) $k^{*}$ is strictly decreasing function in (0, *L*/2),
(iii) $$0< k^{*}(x) < D \qquad (x \in I)$$.

We prove these lemmas in “Appendix D”.

#### Proof of Theorem 4.1

Following Lemmas 4.1 and 4.2, $k^*$ is represented by $k^{*}={\mathcal {H}}^{-1}(-p^{*})$, and from (iii) of Lemma 4.2, the constant $c_f$ is estimated by

$$\begin{aligned} c_f= 1 - \dfrac{1-D}{D}\langle k^{*} \rangle >1 - \dfrac{1 - D}{D}D=D. \end{aligned}$$

Consequently, $c_f \ne 0$. Thus, we can apply *Sherman–Morrison–Woodbury formula* for (25) and obtain

$$\begin{aligned} w_{f}^{*}(x)&= \left\{ 1 + \frac{1-D}{c_f D}\langle k^* \rangle \right\} k^*(x) =\dfrac{D}{D-(1-D)\langle k^{*} \rangle }k^*(x),\\ w_{s}^{*}(x)&= 1 + \dfrac{1-D}{D}\langle {w}_{f}^{*} \rangle -\dfrac{1}{D}{w}_{f}^{*}(x)=\dfrac{D-k^{*}}{D-(1-D)\langle k^{*}(x) \rangle },\\ w^{*}(x)&=w_{f}^{*}(x)+w_{s}^{*}(x)=\dfrac{D-(1-D)k^{*}(x)}{D-(1-D) \langle k^{*} \rangle }. \end{aligned}$$

Due to Lemma 4.2, $k^{*}$ is positive and strictly decreasing function, and $D-(1-D)\langle k^{*}\rangle > D^2$, because of $\langle k^* \rangle <D$. Thus, $w_f^*$ and $w_s^*$ satisfy (i)$\sim$(iii). Moreover, we can prove that $w^*$ satisfies same properties of $w_s^*$ with same manners, when $0<D<1$ . Therefore, the theorem is proved. $\square$

#### Remark 4.3

**Fig. 6:**
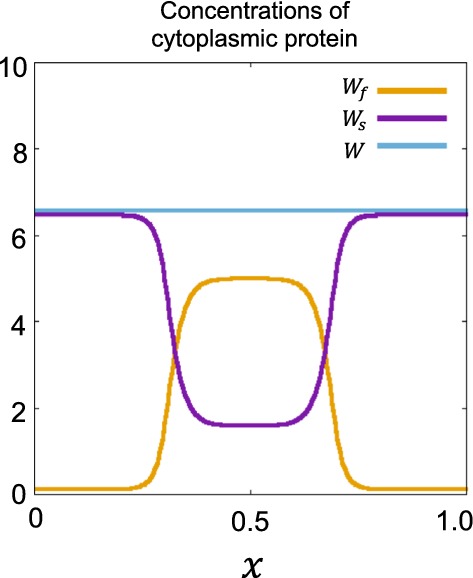
Example of $D_s \approx D_f$ case in TFD model. Representative parameter set Table 2 were chosen except for $D_s=D_f=0.0032$

Let us define $w(x;\xi ):=w_s(x;\xi )+w_f(x;\xi )$. From Theorem 4.1, we directly obtain that *w* is strictly increasing function in (0, *L*/2) if $\xi >0$ and $D<1$. This implies that *w* is anti-phase with respect to $u^*$. However, if $D=1$, then *w* becomes a constant function. Thus, the polarity in the down-stream is not formed, although each $w_s$ and $w_f$ forms a polarity as shown in Fig. 6. This result proves that the difference of diffusion coefficients causes the polarity of the total concentration of downstream proteins.

### The stability of the solution of Eq. (18)

In this subsection, we prove the stability of stationary solutions of the system (18) given by $\widetilde{S}:=(u^*,v^*,w_s^*,w_f^*)$. Let $\widetilde{{\mathcal {L}}}$, $dom(\widetilde{{\mathcal {L}}})$ and $\lambda$ be linearized operators of the right hand side of the system (18) at $\widetilde{S}$, domain of $\widetilde{{\mathcal {L}}}$, and eigenvalue of $\widetilde{{\mathcal {L}}}$.

27 $$\begin{aligned}&dom(\widetilde{{\mathcal {L}}}) := X \times X,\nonumber \\&\widetilde{{\mathcal {L}}} \varvec{\Phi } =\begin{pmatrix} d_1 &{} 0 &{} 0 &{} 0 \\ 0 &{} d_2 &{} 0 &{} 0 \\ 0 &{} 0 &{} d_s &{} 0 \\ 0 &{} 0 &{} 0 &{} d_f \end{pmatrix} \partial _x^2 \varvec{\Phi } +\begin{pmatrix} {f}^*_u &{} {f}^*_v &{} 0 &{} 0 \\ -{f}^*_u &{} -{f}^*_v &{} 0 &{} 0 \\ {g}^*_u &{} 0 &{} -p^* &{} q^* \\ -{g}^*_u &{} 0 &{} p^* &{} -q^* \end{pmatrix} \varvec{\Phi } = \lambda \varvec{\Phi },\nonumber \\&{g}^*_u := g_u(u^*,w_s^*,w_f^*). \end{aligned}$$

We can find that $\widetilde{S}_x$ is the eigenfunction of $\widetilde{{\mathcal {L}}}$ associated with 0 in the similar way with ${\mathcal {L}}$.

#### Lemma 4.3

0 is simple eigenvalue of $\widetilde{{\mathcal {L}}}$ with the eigenfunction $\widetilde{S}_x$.

#### Proof

Let $Ker(\widetilde{{\mathcal {L}}})$ be kernel space of $\widetilde{{\mathcal {L}}}$. Because 0 is simple eigenvalue of ${\mathcal {L}}$ from Assumption 2, any $\varvec{\Phi } \in Ker(\widetilde{{\mathcal {L}}})$ is given by $(0,0,\phi ,\psi )$ or $(u_x,v_x,\phi ,\psi )$ up to scale, where $\phi$ and $\psi$ are some functions with $(\phi , \psi ) \in X$. If we choose $\varvec{\Phi }=(0,0,\phi ,\psi )$, then $(\phi ,\psi )$ satisfies Eq. (23) with $\langle \phi \rangle + \langle \psi \rangle =0$. From Remark 4.2, $\phi =\psi =0$. Therefore we consider the case of $\varvec{\Phi }=(u_x,v_x,\phi ,\psi )$ only. Suppose $\varvec{\Phi _1}, \varvec{\Phi _2} \in X \times X$ are represented by

$$\begin{aligned} \varvec{\Phi _1} = (u_x, v_x, \phi _1, \psi _1), \quad \varvec{\Phi _2} = (u_x, v_x, \phi _2, \psi _2), \end{aligned}$$

up to scale. Substituting them into (27), each equation of $(\phi _1,\psi _1)$ and $(\phi _2, \psi _2)$ is given to

28 $$\begin{aligned}&\left\{ \begin{array}{l} d_s\dfrac{d^2 \phi _1}{dx^2} +{g}^*_u {u}^*_x- p^*(x)\phi _1 + q^*(x)\psi _1=0, \\ d_f\dfrac{d^2 \psi _1}{d x^2} -{g}^*_u {u}^*_x+ p^*(x)\phi _1 - q^*(x)\psi _1=0. \end{array}\right. \end{aligned}$$

29 $$\begin{aligned}&\left\{ \begin{array}{l} d_s\dfrac{d^2 \phi _2}{dx^2} +{g}^*_u {u}^*_x- p^*(x)\phi _2 + q^*(x)\psi _2=0, \\ d_f\dfrac{d^2 \psi _2}{d x^2} -{g}^*_u {u}^*_x+ p^*(x)\phi _2 - q^*(x)\psi _2=0. \end{array}\right. \end{aligned}$$

Taking the differences between each first equation of (28) and (29), and between each second equation of (28) and (29), we obtain

$$\begin{aligned} \left\{ \begin{array}{l} d_s\dfrac{d^2 \widetilde{\phi }}{dx^2} - p^*(x)\widetilde{\phi } +q^*(x)\widetilde{\psi }=0, \\ d_f\dfrac{d^2 \widetilde{\psi }}{d x^2} + p^*(x)\widetilde{\phi } -q^*(x)\widetilde{\psi }=0, \end{array}\right. \end{aligned}$$

where $\widetilde{\phi }:=\phi _1-\phi _2$, $\widetilde{\psi }:=\psi _1-\psi _2$. Due to $(\widetilde{\phi }, \widetilde{\psi }) \in X$, then $\widetilde{\phi }$ and $\widetilde{\psi }$ are solutions of (23) with $\xi = 0$. Thus, $\widetilde{\phi }\equiv 0, \widetilde{\psi } \equiv 0$ from Remark 4.2. Consequently, we obtain $\varvec{\Phi _1} = \varvec{\Phi _2}$. This means any eigenfunction associated with 0 is given by $\widetilde{S}$ up to scale. Moreover, suppose that there is function $\varvec{\Psi } \in X \times X$ such that $\widetilde{{\mathcal {L}}}\varvec{\Psi }=\varvec{\Phi }$, then 0 is not eigenvalue of ${\mathcal {L}}$. This is contradiction because of Assumption 2. Therefore, 0 is simple eigenvalue of $\widetilde{{\mathcal {L}}}$. $\square$

Next, we prove there exist no eigenvalue with positive real part. For the proof, we have referred the mathematical technique in Lemma 2.5 of Bates and Chen (2002), Protter and Weinberger (1984).

#### Theorem 4.2

Any eigenvalue of $\widetilde{{\mathcal {L}}}$ has a negative real part except for 0. Moreover, there is no pure imaginary eigenvalue of $\widetilde{{\mathcal {L}}}$.

#### Proof

(Case 1) The case that $\lambda$ is complex number.

Suppose that $$\lambda =a + b\sqrt{-1}$$$$(a \ge 0, b \ne 0)$$ is eigenvalue of $$\widetilde{{\mathcal {L}}}$$, and we will show a contradiction. From Assumption 2, $$\lambda $$ is not eigenvalue of $${\mathcal {L}}$$. Then, the eigenfunction $$\varvec{\Phi }$$ is given by $$\varvec{\Phi }(x)=^{T}(0,0,\phi _1(x),\phi _2(x))$$. Substituting $$\varvec{\Phi }$$ into Eq. (27), we obtain

30 $$\begin{aligned} \lambda \left( \begin{array}{c} \phi _1 \\ \phi _2 \end{array}\right) =\left( \begin{array}{c} d_s \dfrac{d^2\phi _1}{dx^2} -p^*(x)\phi _1 + q^*(x)\phi _2 \\ d_f \dfrac{d^2\phi _2}{dx^2} +p^*(x)\phi _1 - q^*(x)\phi _2 \end{array}\right) =:{\mathcal {L}}^{\dagger } \left( \begin{array}{c} \phi _1\\ \phi _2 \end{array}\right) _{,} \end{aligned}$$

where $(\phi _1(x),\phi _2(x))=(\varphi _1(x),\varphi _2(x))+ \sqrt{-1}(\psi _1(x),\psi _2(x))$ and each of $\varphi _1,\varphi _2, \psi _1, \psi _2$ is real valued function. Let $\Xi (x,t)$ be given function as follows.

$$\begin{aligned} \Theta (x,t):=\begin{pmatrix} \theta _1(x,t) \\ \theta _2(x,t) \end{pmatrix} =\cos (bt)\begin{pmatrix} \varphi _1(x) \\ \varphi _2(x) \end{pmatrix} -\sin (bt)\begin{pmatrix} \psi _1(x) \\ \psi _2(x) \end{pmatrix}_{,} \end{aligned}$$

then, $$\Theta (x,t)$$ satisfies

31 $$\begin{aligned}&\Theta _t = {\mathcal {L}}^{\dagger }\Theta - a\Theta , \quad \theta _1(x,t) \le |\phi _1(x)|,\ \theta _2(x,t) \le |\phi _2(x)| \ \ (x \in I, t > 0), \nonumber \\&\Theta (x,0) = \begin{pmatrix} \varphi _1(x) \\ \varphi _2(x) \end{pmatrix}\ \ (x \in I). \end{aligned}$$

Next, let $\tau _1$ and $\tau _2$ be

$$\begin{aligned} \tau _1&:= \min \{ \tau \in {\mathbb {R}} ; \tau w_{s}^{*}(x) -|\phi _1(x)| \ge 0, \ x \in I\}, \\ \tau _2&:= \min \{ \tau \in {\mathbb {R}} ; \tau w_{f}^{*}(x) -|\phi _2(x)| \ge 0, \ x \in I\}, \end{aligned}$$

where $\tau _1>0$ or $\tau _2>0$, because $w_s^*, w_f^*>0$ and $\phi _1 \not \equiv 0$ or $\phi _2 \not \equiv 0$. We consider the case $\tau _1 \ge \tau _2$. From the definition of $\tau _1$, there exist $x_0 \in I$ such that $\tau _1 w_{s}^{*}(x_0) = |\phi _1(x_0)|$.

We define $W(x):=^{T}(w_{s}^{*}(x),w_{f}^{*}(x))$ and let *V*(*x*, *t*) be the function given by

$$\begin{aligned} V(x,t) := \begin{pmatrix} v_1(x,t) \\ v_2(x,t) \end{pmatrix} = \tau _1W(x) -\Theta (x,t). \end{aligned}$$

From $\tau _1 \ge \tau _2$ and $\Theta (x,t)$ satisfies (31), *V* satisfies $V(x,t) \ge 0 \ (x \in I, t>0)$ and $V(x,0) \not \equiv 0$ hold. Moreover,

$$\begin{aligned} V_t - {\mathcal {L}}^{\dagger }V+aV&= -\Theta _t + {\mathcal {L}}^{\dagger }\Theta +aV=-({\mathcal {L}}^{\dagger }\Theta -a\Theta ) + {\mathcal {L}}^{\dagger }\Theta +aV\\&=a\Theta +aV= a\tau _1W \ge 0. \end{aligned}$$

Therefore, applying strong comparison principle (Protter and Weinberger 1984), we obtain $V(x,t)> 0\ (x\in I, t>0)$. However, in the first component of $\Theta (x,t)$, there exists $t_0>0$, such that

$$\begin{aligned} \cos (bt_0)\varphi _1(x_0)-\sin (bt_0)\psi _1(x_0)=|\phi _1(x_0)|. \end{aligned}$$

Then, we obtain

$$\begin{aligned} 0 < v_1(x_0,t_0) = \tau _1 w^{*}_s(x_0) - |\phi _1(x_0)| =0. \end{aligned}$$

This is contradiction. We can prove in the case $\tau _1 \le \tau _2$ by similar manner, thus $\lambda$ is not eigenvalue.

(Case 2) The case that $\lambda$ is real number.

Suppose that $\lambda >0$ is real eigenvalue of $\widetilde{{\mathcal {L}}}$, we will show a contradiction. In the same way of (Case 1), the corresponding eigenfunction is given by $(0,0,\varphi _1(x),\varphi _2(x))$. In here, $\varphi _1, \varphi _2$ are real valued functions and let us suppose that there exist some $x^* \in I$, such that $\varphi _1(x^*)>0$ or $\varphi _2(x^*)>0$.

Next, let $\tau _1$ and $\tau _2$ be

$$\begin{aligned}&\tau _1 := \min \{ \tau \in {\mathbb {R}} ; \tau w_{s}^{*}(x) - \varphi _1(x) \ge 0,\ x \in I\}, \\&\tau _2 := \min \{ \tau \in {\mathbb {R}} ; \tau w_{f}^{*}(x) - \varphi _2(x) \ge 0,\ x \in I\}, \end{aligned}$$

where $\tau _1>0$ or $\tau _2>0$, because $w_{s}^{*},w_{f}^{*}>0$. Now, we consider the case $\tau _1 \ge \tau _2$, and let us define a function *V*(*x*) as follows.

$$\begin{aligned} V(x):= \begin{pmatrix} v_1(x) \\ v_2(x) \end{pmatrix} = \begin{pmatrix} \tau _1w_{s}^{*}(x) - \varphi _1(x) \\ \tau _1 w_{f}^{*}(x) - \varphi _2(x) \end{pmatrix}=\tau _1 W(x) - \Phi (x), \end{aligned}$$

where $v_1(x) \ge 0$ and $v_2(x) \ge 0 \ \ (x \in I)$. From the definition of $\tau _1$, there exist $x_0\in I$, such that $v_1(x_0) = 0$. Moreover, it is hold that

$$\begin{aligned} {\mathcal {L}}^{\dagger }V(x) - \lambda V(x)&= {\mathcal {L}}^{\dagger } (\tau _1 W(x) - \Phi (x)) - \lambda (\tau _1W(x) - \Phi (x))\\&=-\lambda \tau _1 W(x). \end{aligned}$$

For the first component of above equation at $x=x_0$, we obtain

$$\begin{aligned} 0 > -\lambda \tau _1 w_{s}^{*}(x_0)&= d_s \dfrac{d^2v_1}{dx^2}(x_0) -p(x_0)v_1(x_0) + q(x_0)v_2(x_0) -\lambda v_1(x_0)\\&\ge d_s \dfrac{d^2v_1}{dx^2}(x_0) + q(x_0)v_2(x_0) \ge 0, \end{aligned}$$

because $v_1$ takes minimal value 0 at $x=x_0$. This is contradiction. Similarly, we can also prove for the case of $\tau _1 \le \tau _2$. Therefore, $\lambda$ is not eigenvalue.

From (Case1) and (Case2), the theorem is proved. $\square$

## Discussion

In the past decade, both theoretical and experimental approaches for elucidating polarity formation in a single cell have been extensively explored (Knoblich 2008; Otsuji et al. 2007; Mori et al. 2011; Hoege and Hyman 2013; Trong et al. 2014; Seirin-Lee and Shibata 2015; Seirin-Lee 2016; Campanale et al. 2017; Kuwamura et al. 2018). However, most studies have focused on the polarity occurring in the cell membrane, and the cytoplasmic polarity has been poorly understood. In this study, we focused on finding an essential mechanism of cytoplasmic polarity and polarity positioning between membrane and cytoplasmic proteins, a phenomenon that is motivated by asymmetric cell division of *C. elegans* embryo. We devised a conceptional model for downstream polarity formation with respect to upstream polarity formation by capturing the essential dynamics of proteins in *C. elegans* embryo upstream of membrane protein (PARs) and downstream of cytosol proteins (MEX-5/6 and PIE-1). Using the conceptional model, we found a general mechanism by which the proteins upstream of membrane proteins determine the relative downstream position of cytosol proteins. Especially, we found that the mechanism for the downstream polarity to be in an anti-phase pattern is based on the transitional regulation such as *activation* from slow type to *fast type* and the *inhibition* from fast type to *slow type* by an upstream polarity substrate (TFD model). In contrast, the mechanism for the downstream polarity to be in an in-phase pattern involves the reverse regulation of the anti-phase mechanism (TSD model). This result shows that the mechanism for the fast/slow type transition to be promoted plays a key role in inducing anti-phase/in-phase polarity. We also found that these mechanisms are mathematically generalized and confirmed that they hold under some general mathematical conditions.

For generating downstream polarity, we found that both establishment and maintenance of upstream polarity are indispensable and that a proper proportion of conversion rates is essential. These results imply that the heterogeneous transition of diffusive type of downstream proteins is important for downstream polarity (Wu et al. 2018). Indeed, our mathematical analysis shows that there exists a non-constant solution of downstream proteins under these conditions (Assumptions 1). In addition, we also numerically found and mathematically confirmed that the difference in diffusion rates between the fast type and slow type transition is indispensable. In this case, downstream polarity does not appear, although the fast type and slow type transitions generate clear polarities (Remark 4.3). In contrast, from the comparison of numerical results between TFD and TSD models for the parameter spaces (the difference of diffusion rates between fast type and slow type and the balance of conversion rates) with respect to the existence of downstream polarity, we found that the parameters do not affect the location of downstream polarity. Indeed, our mathematical proof does not require a different set of conditions for the TFD and TSD models (Lemma 4.1). From these results, we conclude that the mathematical structure to form the downstream polarity itself is independent of the details of the mechanism of polarity location, and the polarity positioning can be completely determined by the properties of regulation by upstream protein on the downstream protein.

Finally, we constructed phenomenological models for the polarity proteins, PARs, MEX-5/6, and PIE-1, in *C. elegans* and confirmed that the multiple streams polarity dynamics in *C. elegans* can be understood by our two conceptional models. This implies that the mechanism for the relative positioning between upstream and downstream polarity which we found here may become a general mechanism to explain the position of multiple streams of polarity dynamics in asymmetric cell division. Furthermore, our mathematical analysis under general conditions suggests that such a mechanism can be understood without specific bio-chemical reactions or phenomena and thus can be a universal mechanism for polarity positioning without the requirement of cell specification.

## A: Reduction to self-recruitment model

One-dimensional $(U_1, V_1)$ and $(U_2, V_2)$ polarity model (Seirin-Lee and Shibata 2015) is given to

32 $$\begin{aligned}&\frac{\partial u_1}{\partial t}=D_{m_1}\frac{\partial ^2 u_1}{\partial x^2} +\gamma v_1-f_1(u_2)u_1, \nonumber \\&\frac{\partial v_1}{\partial t}=D_{c_1}\frac{\partial ^2 v_1}{\partial x^2} -\gamma v_1+f_1(u_2)u_1,\nonumber \\&\frac{\partial u_2}{\partial t}=D_{m_2}\frac{\partial ^2 u_2}{\partial x^2} +\bar{\gamma } v_2-f_2(u_1)u_2, \nonumber \\&\frac{\partial v_2}{\partial t}=D_{c_2}\frac{\partial ^2 v_2}{\partial x^2} -\bar{\gamma } v_1+f_2(u_1)u_2, \end{aligned}$$

where

$$\begin{aligned} f_1(u_2)=\alpha +\frac{K_1 u_2^2}{K+u_2^2}, \qquad f_2(u_1) =\bar{\alpha }+\frac{\bar{K}_1 u_1^2}{\bar{K}+u_1^2}. \end{aligned}$$

We assume that the polarity of $U_1$ and $U_2$ in the membrane is established from the initial situation where $U_2$ is distributed uniformly in the membrane, in which a small amount of $U_1$ invades at some point of the membrane from the cytosol. We assume sufficiently fast diffusion in the cytosol so that the concentration of $V_1$ and $V_2$ are quickly homogeneous and keep an equilibrium state (denoted by $v_1^*$ and $v_2^*$, respectively). This assumption can be rewritten as the following mathematical conditions:

33 $$\begin{aligned}&\bar{\gamma } v_2^*-f_2(u_1)u_2 \approx 0, \nonumber \\&|u_1(x, t)| \ll 1. \end{aligned}$$

Using Taylor expansion, we can approximate $f_2(u_1)$ such that

34 $$\begin{aligned} f_2(u_1)=\bar{\alpha } +\frac{\bar{K_1}u_1^2}{\bar{K} +u_1^2} \approx \bar{\alpha } + \bar{\beta } u_1^2+O(u_1^3) \end{aligned}$$

where $\bar{\beta }=\bar{K}_1/\bar{K}$. Substituting (34) into Eq. (33), we obtain

35 $$\begin{aligned} u_2\approx \frac{\bar{\gamma } v_2^*}{\bar{\alpha } +\bar{\beta } u_1^2}=\frac{\delta _2}{1+\delta _1 u_1^2}, \end{aligned}$$

where $\delta _1=\bar{\beta }/\bar{\alpha }$ and $\delta _2=\bar{\gamma } v_2^*/\bar{\alpha }$. Then, we have

36 $$\begin{aligned} f_1(u_2)&=\alpha +\frac{K_1 u_2^2}{K+u_2^2}\approx \alpha + \frac{K_1}{K\left( \frac{1 + \delta _1 u_1^2}{\delta _2} \right) ^2+1} \nonumber \\&=\alpha +\frac{K_1}{1+\frac{K}{\delta _2^2} + \frac{2\delta _1 K}{\delta _2^2}u_1^2 + O(u_1^3)}\approx \alpha +\frac{\beta _2}{1+\beta _1 u_1^2}, \end{aligned}$$

where $$\beta _1=\frac{2 \delta _1 K}{\delta _2^2}/\left( 1+\frac{K}{\delta _2^2}\right) $$ and $$\beta _2=K_1/\left( 1+\frac{K}{\delta _2^2}\right) $$.

Now, we substitute (36) into the equation of $(u_1, v_1)$ in (32), then the self-recruitment model is given to

$$\begin{aligned}&\frac{\partial u_1}{\partial t}=D_{m_1}\frac{\partial ^2 u_1}{\partial x^2} +\gamma v_1-\left( \alpha +\frac{\beta _2}{1+\beta _1 u_1^2} \right) u_1, \\&\frac{\partial v_1}{\partial t}=D_{c_1}\frac{\partial ^2 v_1}{\partial x^2} -\gamma v_1+\left( \alpha +\frac{\beta _2}{1+\beta _1 u_1^2} \right) u_1. \end{aligned}$$

In addition, from Eq. (35), *G*(*x*, *t*) in Type I and II is given to

$$\begin{aligned} \text {TFD: } G({x}, t)&=\mu _1 u_1({x}, t) w_s({x}, t) -\mu _2 \frac{\delta _2}{1+\delta _1 u_1(x, t)^2} w_f({x}, t), \\ \text {TSD: } G({x}, t)&=\mu _1 \frac{\delta _2}{1+\delta _1 u_1(x,t)^2} w_s({x}, t)-\mu _2 u_1({x}, t) w_f({x}, t). \end{aligned}$$

Note that the reduction has been carried under the assumption of $|u_1(x, t)| \ll 1$. However, we have found that the polarity dynamics in the reduced model is essentially similar with the original model as shown in Fig. 2.

## B: Initial conditions and detailed parameters for simulations

In this paper, we used a local concentration type, which represents that a cell polarity starts with an auxiliary mechanism in a cell or by a strong external stimulation, such as an invasion of sperm in *C. elegans* embryo cell, in which the anterior protein initially dominates a whole membrane and the posterior protein is distributed only in the cytosol (Gönczy 2005). Thus, we give the initial condition for $U_1$ or *U* such that

$$\begin{aligned} u(x, 0)=u_0 \delta (x-L/2), \end{aligned}$$

where $u_0$ is given, and for the other substrates, for example *v*, such that

$$\begin{aligned} v(x,0)=\frac{v_0}{L}(1+\epsilon \phi _c (x)) \quad \text {for}\quad x\in [0,~L], \end{aligned}$$

where $v_0$ is a homogeneous steady states obtained from the model equation with a given $u_0$ and $\phi _c(x)$ are perturbation functions and we take $\epsilon$ in [0.001, 0.01].

For simulations, we chose parameter values for spatial size, time scale and diffusion coefficients based on the experimental data from the *C. elegans* embryo (Goehring et al. 2011; Daniels et al. 2009; Wu et al. 2018). The representative parameter set is given in Table 2 and the detailed parameter values used in each figure are given below.

- **Figure** 2 (A) $u_{10}=0.26, ~u_{20}=0.56, ~w_{f0}=0.4, ~K_1=1.2, ~\bar{K}_1=5.2,~K=0.8,~\bar{K}=1.25, ~\alpha =0.06, ~\bar{\alpha }=0.06,~\gamma =0.2,~\bar{\gamma }=0.2$ for both I and Type II. $\mu _1=1.0,~\mu _2=0.2$ for Type I and $\mu _1=1.0,~\mu _2=1.0$ for Type II. (B) $u_{0}=0.26, ~w_{f0}=0.4, ~\beta _1=4.5,~\beta _2=4.0,~\alpha =0.06, ~\gamma =0.2$ for both TFD and TSD. $\mu _1=1.0,~\mu _2=0.2, \delta _1=0.5, \delta _2=10.0$ for TFD and $\mu _1=0.1,~\mu _2=1.0, \delta _1=0.5, \delta _2=10.0$ for TSD.
- **Figure** 3 (A) Same parameter values with Fig. 2(B) TFD except for $u_{0}=0.16$. (B) Same parameter values with Fig. 2(B) TFD except for $u_{0}=0.56$ and $D_m=D_f=2.40 \times 10^{-5}$. (C–D) $u_{0}=0.56, ~w_{f0}=0.4, ~\beta _1=4.5,~\beta _2=4.0,~\alpha =0.06, ~\gamma =0.2, \delta _1=0.5, \delta _2=10.0$.
- **Figure** 4 (B) $[P_{m}]_0=0.56, ~[M_{f}]_0=0.4,~[PI_f]_0=1.0, ~\beta _1=4.5,~\beta _2=4.0,~\alpha =0.06, ~\gamma =0.2, \delta _1=0.5, \delta _2=10.0, \mu _1=1.0,~\mu _2=0.2, \gamma _1=1.0,~\gamma _2=5.0, \gamma _3=5.0.$ (C) The case of Ca: Same parameter values with (B). The case of Cb: Same parameter values with (B) except for $D_s$. $M_f^*$ was found by calculating the model (11)–(12) with sufficiently large $D_f$ and $D_s=0$, and we obtained spatially homogeneous solution about $M_f^*=0.19209$. (D) Same parameter values with (B). (E) Same parameter values with (B) except for $D_s$. $M_f^*=0.19209$.

**Table 2:** Representative parameter set

Parameter	Dimensional value	Dimensionless value
*L*	$142.75\ \upmu \hbox {m}$	1.0
*t*	$4.0 ~\hbox {s}$	1.0
$D_{m1}$, $D_m$	$0.122\ \upmu \hbox {m}^2/\hbox {s}$	$2.40 \times 10^{-5}$
$D_{c1}$, $D_c$	$7.743\ \upmu \hbox {m}^2/\hbox {s}$	$1.44 \times 10^{-3}$
$D_{m2}$	$0.122\ \upmu \hbox {m}^2/\hbox {s}$	$2.40 \times 10^{-5}$
$D_{c2}$	$7.743\ \upmu \hbox {m}^2/\hbox {s}$	$1.44 \times 10^{-3}$
$D_{s}$	$0.65\ \upmu \hbox {m}^2/\hbox {s}$	$1.28 \times 10^{-4}$
$D_{f}$	$16.3\ \upmu \hbox {m}^2/\hbox {s}$	$3.20 \times 10^{-3}$
$D_{ps}$	$0.6\ \upmu \hbox {m}^2/\hbox {s}$	$1.180 \times 10^{-4}$
$D_{pf}$	$8.7\ \upmu \hbox {m}^2/\hbox {s}$	$1.708 \times 10^{-3}$

## C: Additional figures

Figure 6 shows that the concentration *W* shows homogeneous state although $W_s$ and $W_f$ generate clear polarities when there is little difference in diffusion rates between $D_s$ and $D_f$.

## D: Proof of Lemmas 4.1 and 4.2

### D.1: Proof of Lemma 4.1

(Uniqueness.) Suppose that $k_1$ and $k_2$ are solutions of (26). Substituting each of $k_1$ and $k_2$ to (26), and taking the difference between each equation, we obtain

$$\begin{aligned} \dfrac{d^2\tilde{k}}{dx^2}-\rho (x)\tilde{k}=0, \end{aligned}$$

where $\tilde{k}:=k_1-k_2$. Multiplying both side of above equation by $\tilde{k}$, and integrating it for the interval *I*, we obtain

$$\begin{aligned} \int _I \dfrac{d^2\tilde{k}}{dx^2}\tilde{k}-\rho (x)\tilde{k}^2 dx&=\left[ \dfrac{d\tilde{k}}{dx}\tilde{k} \right] _{x=-L/2}^{x=L/2} - \int _I\left( \left( \dfrac{d\tilde{k}}{dx}\right) ^2+\rho (x)\tilde{k}^2\right) dx\\&=- \int _I\left( \left( \dfrac{d\tilde{k}}{dx}\right) ^2+\rho (x)\tilde{k}^2\right) dx = 0, \end{aligned}$$

accordingly, $\tilde{k}\equiv 0$, then $k_1 = k_2$. This means the uniqueness of solutions.

(Existence) By the uniqueness of solution, if the solution exists, it must be even function and satisfies $\dfrac{dk^*}{dx}(0)=\dfrac{dk^*}{dx}(\pm L/2)=0$. Thus, we consider the existence of solution for the equation as follows.

37 $$\begin{aligned} \left\{ \begin{array}{l} {\mathcal {H}} k = -p^*(x), \qquad x \in I_{+}, \\ \dfrac{dk}{dx}(0)=\dfrac{dk}{dx}(L/2)=0, \end{array}\right. \end{aligned}$$

where $I_{+}:=(0,L/2)$, ${\mathcal {H}}:=\dfrac{d^2}{dx^2} -\rho (x)$. This equation is Sturm–Liouville equation and any eigenvalue of ${\mathcal {H}}$ is real number. Let $\lambda _0$ be maximal eigenvalue of ${\mathcal {H}}$ and $\Phi$ be the eigenfunction associated with $\lambda _0$, then $\lambda _0$ is given by (Dunford and Schwartz 1988).

$$\begin{aligned} \lambda _0=\sup _{k \in {H}^1(I_{+}), k \not \equiv 0} \dfrac{- \displaystyle \int _{I_{+}}\left( \left| \dfrac{dk}{dx}\right| ^2 +\rho (x)k^2\right) dx}{\displaystyle \int _{I_{+}}k^2dx} =\dfrac{- \displaystyle \int _{I_{+}}\left( \left| \dfrac{d\Phi }{dx}\right| ^2 +\rho (x)\Phi ^2\right) dx}{\displaystyle \int _{I_{+}}\Phi ^2dx}<0, \end{aligned}$$

because $\rho >0$. This means 0 is element of the resolvent set of ${\mathcal {H}}$, then there exists inverse operator ${\mathcal {H}}^{-1}$, therefore (37) has a unique solution $k_N$. Now, we let $k^*$ given function as follows.

$$\begin{aligned} k^{*}(x) :=\left\{ \begin{array}{ll} k_N(x)&{}\quad (x \in (0,L/2)),\\ k_N(-x) &{}\quad (x \in (-L/2,0)). \end{array}\right. \end{aligned}$$

It is clear that $k^{*}$ is solution of (26). Therefore, the existence of solution is proved. $\square$

#### Remark

When we suppose Assumption 1 and 3, Eq. (21) can be written as the following

38 $$\begin{aligned}&\left\{ \begin{array}{l} \dfrac{\partial w_s}{\partial t}=D\dfrac{d^2 w_s}{dx^2} - p^*(x)w_s + q^*(x)w_f, \\ \dfrac{\partial w_f}{\partial t}=\dfrac{d^2 w_f}{dx^2} + p^*(x)w_s - q^*(x)w_f,\\ \end{array} \right. \qquad x \in I.\nonumber \\&p^*(x):=p(u^*(x)),\quad q^*(x):=q(u^*(x)). \end{aligned}$$

This equations have a conserved quantity and the comparison principle is holed under the periodic boundary conditions (Protter and Weinberger 1984). Thus, we can also directly prove the existence and uniqueness of the stationary solutions of Eq. (38) from the result of Ogiwara (2014) in which more general type of equations was considered.

### D.2: Proof of Lemma 4.2

Proof of (i) is already done in the proof of Lemma 4.1, then we prove (ii) and (iii).

(Proof of (ii)) First, we prove $\dfrac{dk^{*}}{dx}(x) \le 0\ (x \in I_{+})$. Suppose $k^*$ doesn’t satisfy this condition, and we will show a contradiction. By Lemma 4.1, the derivative of $k^{*}$ takes 0 at $x=0, K/2$. Then there exist $x_1 \in I_{+}$ and positive constant $\delta _1=\delta _1(x_1)$, such that

39 $$\begin{aligned}&\dfrac{dk^{*}}{dx}(x_1)> 0, \quad \dfrac{d^2 k^{*}}{dx^2}(x_1) = 0,\nonumber \\&\dfrac{dk^{*}}{dx}(x) > 0,\quad \dfrac{d^2k^{*}}{dx^2}(x) < 0,\quad (x \in I_{\delta _1} \subset I_{+}), \end{aligned}$$

where $I_{\delta }:=(x_1, x_1+\delta )$ for $\delta > 0$. In the view of (26),

40 $$\begin{aligned} \dfrac{d^2 k^{*}}{dx^2}(x)<0&\Leftrightarrow \rho (x)k^{*}(x)-p^*(x)<0,\nonumber \\&\Leftrightarrow k^{*}(x) < \dfrac{p^*(x)}{\rho (x)} =: \tilde{\rho }(x), \end{aligned}$$

therefore, $k^{*}(x)<\tilde{\rho }(x) \ (x \in I_{\delta _1})$. From Assumption 1 and (*TFD*), $\tilde{\rho }$ is strictly decreasing function in $I_{+}$, then $k^{*}(x)<\tilde{\rho }(x_1)\ (x \in I_{\delta _1})$. However, due to (39), there exists a constant $\delta _2=\delta _2(x_1)>0$, such that

$$\begin{aligned} k^*(x_1)=\tilde{\rho }(x_1) \quad k^{*}(x) >\tilde{\rho }(x_1) \quad (x \in I_{\delta _2} \subset I_{+}). \end{aligned}$$

Let $\delta _3 := \mathrm{min} \{ \delta _1, \delta _2 \}$, then $k^{*}(x) < \tilde{\rho }(x_1)$ and $k^{*}(x)>\tilde{\rho }(x_1)\quad (x \in I_{\delta _3})$. This is contradiction, then $\dfrac{dk^{*}}{dx}(x) \le 0\ (x \in I_{+})$. This means $k^*$ is decreasing function for $x \in I_{+}$.

Next, we prove that $k^*$ is strictly increasing in $I_{+}$. Suppose $k^*$ doesn’t satisfy it, then there exists open interval $(a,b) \subset I_{+}$, such that $\dfrac{dk^{*}}{dx}(x)=0$ for $x \in (a,b)$. Therefore $k^*$ is constant in (*a*, *b*), then $\dfrac{d^2 k^{*}}{dx^2}(x) = 0$. In view of (40), $k^{*}(x)=\tilde{\rho }(x)$ for $x \in (a,b)$, then $\tilde{\rho }(x)$ is constant in (*a*, *b*). This is contradiction.

(Proof of (iii)) Due to Lemma 4.2, $k^{*}$ is even and strictly decreasing function in *I* and $I_{+}$, then we only have to prove that $k^*(0)<D, k^*(K/2)>0$. Because $k^{*}$ takes maximum at $x=0$, $\dfrac{d^2k^{*}}{dx^2}(0)\le 0$ and $k^{*}(0) \le \tilde{\rho }(0)<D$, due to (40). It is proved that $k^*(K/2)>0$ by same manners. $\square$
